# Thermal Aging Evaluation of XLPE Power Cable by Using Multidimensional Characteristic Analysis of Leakage Current

**DOI:** 10.3390/polym14153147

**Published:** 2022-08-02

**Authors:** Yong Liu, Hao Wang, Han Zhang, Boxue Du

**Affiliations:** Tianjin Key Laboratory of Smart Energy and Information Technology, School of Electrical and Information Engineering, Tianjin University, Tianjin 300072, China; wang_haoovo@163.com (H.W.); zh605279246@163.com (H.Z.); duboxue@tju.edu.cn (B.D.)

**Keywords:** XLPE cable, thermal aging, leakage current, harmonic characteristics, feature extraction

## Abstract

Thermal aging is a common form of cable deterioration. In this paper, the effect of thermal aging on cables is evaluated by analyzing the harmonic characteristics in cable leakage currents. Cable samples were first fabricated and subjected to accelerated thermal aging tests at 120 °C. The experimental circuits were built to test the dielectric loss factor and the AC leakage current of the cable at different aging times. Then, the improved variational modal decomposition (VMD) algorithm was used for the time–frequency analysis of the leakage current, and the relationship between thermal aging and leakage current harmonics was investigated. Thermal aging was discovered to increase the capacitance and dielectric loss factor of the cable as well as generate harmonics in the leakage current, with harmonics at 150, 450, and 650 Hz being particularly sensitive to thermal aging. The multidimensional characteristic parameters such as the time-domain, frequency-domain, and relative energy and the sample entropy of the leakage current harmonics were calculated. The results demonstrated thermal aging increased the relative energy and power spectrum energy of the harmonics and increased the disorder of the harmonic sequence.

## 1. Introduction

With the development of urbanization and the increasing demand for reliability of power supply, XLPE cables have been widely used in power networks because of their excellent electrical performance, simple laying, and easy maintenance. Usually, the service life of an XLPE cable is 20–30 years. However, with the extension of operation years, the problems in the operation and maintenance process of the cable are gradually exposed. The cables that were put into service earlier have started to show aging and deterioration [1,2]. Among them, thermal aging is considered as one of the most severe factors leading to the degradation of the insulation performance of cables [3]. Therefore, it is important to monitor the cable operation status, especially the thermal aging condition, to improve the stability of the power supply system. 

Thermal aging is a typical form of cable deterioration, and many scholars have studied the mechanism of thermal aging. Kim C et al. [4] investigated the effect of thermal aging on the insulation performance of XLPE and discovered that the insulation break-down strength dropped as the aging period increased, while thermal aging caused a breaking of the molecular chain structure. Kemari Y et al. [5] found that temperature can change the crystalline state of XLPE, which leads to a decrease in the dielectric constant. Suo C et al. [6] investigated the thermal aging stages of insulation and discovered that the properties of the insulation did not change much in the first stage, whereas in the second stage, the XLPE microscopic morphology was severely damaged due to crystalline disruption inside the insulation, which reduced the electron binding effect. Andjelkovic D et al. [7] studied the physicochemical properties of XLPE insulation. The results showed that the tensile strength and elongation at break of the XLPE cable’s insulation were affected by the crystallinity under thermal aging conditions, and the trend was the same as that of the crystallinity. Boukezzi L et al. [8] characterized the microstructure of XLPE insulation and found that the high-temperature environment affected the crystallinity of XLPE but did not change the crystalline phase. Mecheri, Y. et al. [9] investigated the changes in dielectric loss and mechanical properties of cross-linked insulation under thermal aging and the test results showed that the dielectric loss of XLPE specimens became larger and the mechanical properties decreased after thermal aging.

Condition monitoring refers to the online monitoring of cable condition quantities to obtain cable condition information. Common methods include DC superposition method, DC component method, dielectric loss angle tangent method [10], partial dis-charge method [11,12,13], grounding current method [14,15,16], etc. The DC superposition method usually involves adding a DC power supply to the cable system, filtering out the AC components, finding the insulation resistance, and evaluating the cable condition based on the insulation resistance value. The DC component method, on the other hand, estimates the degree of deterioration of XLPE-insulated cables by detecting the magnitude of the DC component [17]. Zhu G et al. [18] proposed a novel method based on low-frequency signal injection. This approach calculated the dielectric loss angle of the cable’s leakage current and consequently evaluated the cable insulation. Rizzi A et al. [19] designed an automatic diagnosis system for partial discharges in cable accessories, by selecting the optimal subset of features in the partial discharge information set and then classifying the complex discharge pulse patterns in different power cable terminals and joints. The grounding current method can obtain information on the operating condition of the cable by detecting changes in the grounding wire current. 

In this paper, the relationship between the thermal aging of cables and harmonic characteristics of leakage currents was investigated by a time–frequency domain analysis and feature extraction methods. Cable samples were fabricated and subjected to thermal aging tests at 120 °C. The effects of thermal aging on the dielectric properties of cable insulation were analyzed by capacitance and dielectric loss factor tests. The leakage currents of the cable samples were measured, and the harmonic characteristics of the leakage currents were analyzed using the time–frequency domain method. Furthermore, the time–frequency domain characteristic parameters of the harmonics were extracted, and the distribution of the characteristic parameters was examined. The work done will provide a reference for the insulation evaluation of in-service XLPE cables.

## 2. Samples and Experimentation

### 2.1. Sample Preparation

**Figure 1:**
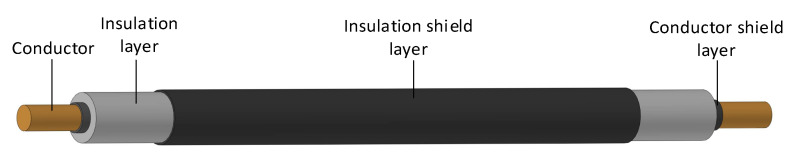
The cable sample.

The experimental cable type was YJLV 8.7/15 kV–1 × 70 mm^2^, with an aluminum conductive core and an XLPE insulation layer, and the nominal thickness of insulation layer was 4.5 mm. Considering that the conductive core may discharge to the insulation shield when an AC high voltage is applied, 60 mm of insulation shield was removed from each end of the sample to maintain sufficient insulation spacing. The cable sample is shown in Figure 1. Due to the size limitation of the air blast drying box, the total length of the fabricated cable sample was 400 mm, of which the length of the insulation shield was 240 mm.

The aging temperature was set to 120 °C. The HK-450A+ electric thermostat blast drying box was applied to realize the thermal aging of the cable. The aging chamber was adjusted to the required aging temperature, the sample was put in after the temperature in the aging chamber reached the set value, and the sample was taken out for testing at the aging time of 12 days, 24 days, and 48 days, respectively.

### 2.2. Experimental Setup

Before conducting the experimental test, we used clean alcohol cotton balls to wipe the cable sample surface, we applied a conductive silver paint on the middle part of the insulation shield of the cable sample, and we wound copper foil as the test electrode.

**Figure 2:**
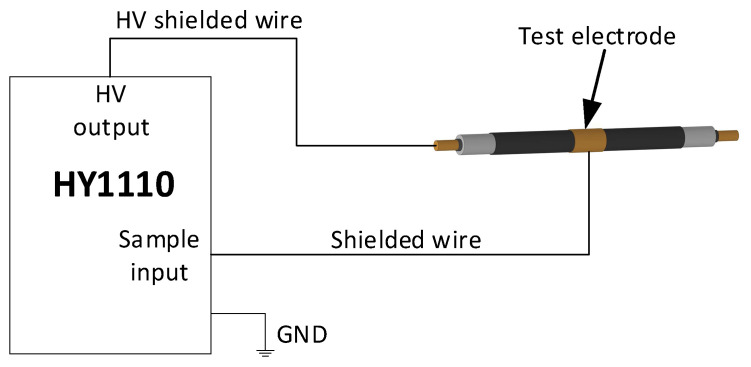
The cable capacitance and dielectric loss factor measurement device.

The HY 1110-type measuring device was used to test the capacitance and dielectric loss factor of the cable. Each cable sample was grounded and discharged before testing. The test wiring is shown in Figure 2. The HV output terminal of the HY1110 was connected to the cable conductor through a HV shield wire, and the sample input terminal of the HY1110 was connected to the test electrode of the cable sample through a shield wire; then, the HY1110 was reliably grounded. After the wiring was completed, the test voltage was set to 3 kV and each cable sample was tested 10 times. After the test of each cable was completed, the cable sample was discharged using a discharge rod, and then the cable sample was replaced for the next test.

**Figure 3:**
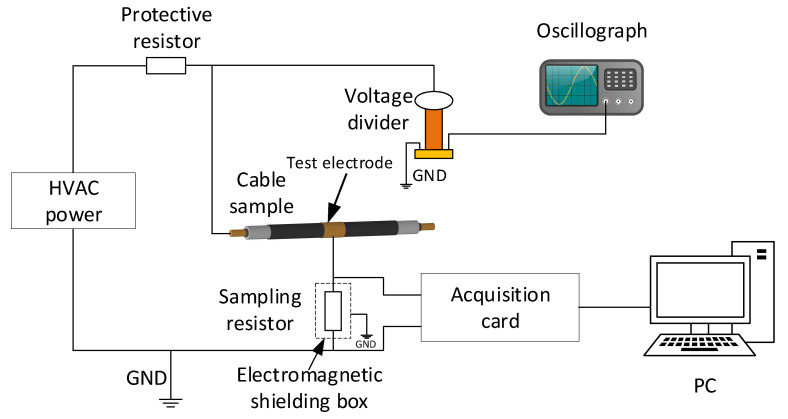
The AC leakage current test experimental setup.

The leakage current test experimental setup is shown in Figure 3. The HVAC power consisted of a voltage regulator and a test transformer, which could generate AC voltages of up to 50 kV. The protection resistor had a resistance value of 1 MΩ to prevent short-circuit overcurrent shocks to the test system. The high-voltage output was connected to the cable sample conductor, while the test electrode of the cable sample was connected to the sampling resistor. The sampling resistor with a resistance value of 10 kΩ, which was placed in an electromagnetic shielding box to reduce external interference, converted the current flowing through the insulation layer into a voltage signal to be collected by the acquisition card and stored on a PC, where the sampling frequency range of the acquisition card was adjustable from 0 to 250 MHz. Meanwhile, the voltage applied to the cable sample during the experiment was 8.7 kV, and this synchronization signal was collected by means of a voltage divider and an oscilloscope.

### 2.3. Experimental Results

**Figure 4:**
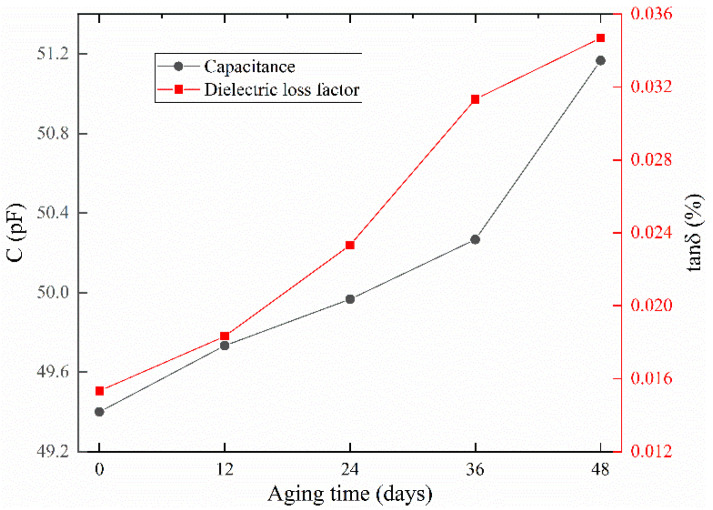
The changes in capacitance and dielectric loss factor of cable sample with aging time.

The change of capacitance and dielectric loss factor of cable sample with aging time is shown in Figure 4. From the figure, it is seen that the capacitance and dielectric loss factor increase gradually with the aging time. After 48 days of aging, the capacitance and dielectric loss factor of the cable sample increase by 3.576% and 126.354%, respectively, compared with those of the unaged sample. Therefore, the thermal aging makes the dielectric properties of XLPE insulation deteriorate and the loss increase.

**Figure 5:**
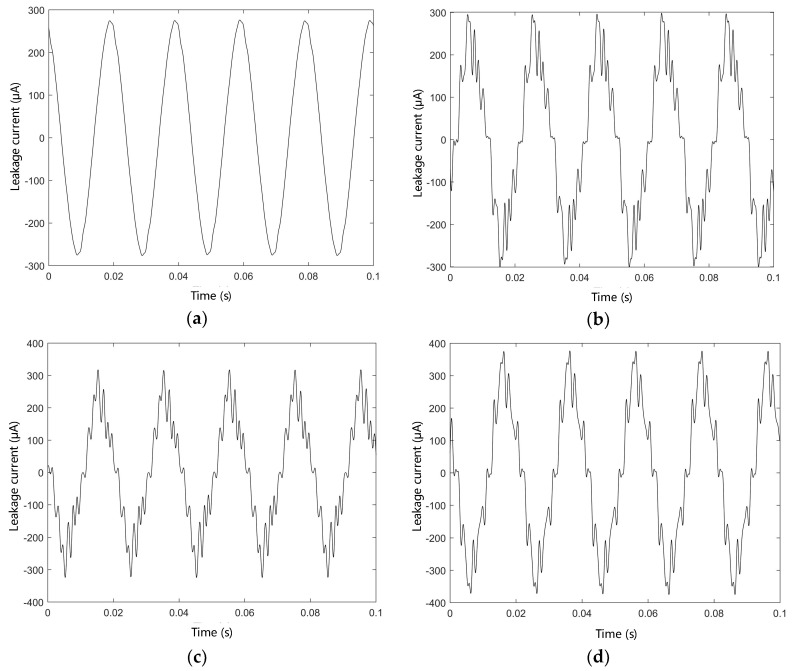
The leakage current test results of cable samples. (**a**) Unaged; (**b**) aging for 12 days; (**c**) aging for 24 days; (**d**) aging for 48 days.

The results of the cable leakage current test are shown in Figure 5. It can be seen from the figure that for the unaged cable, the leakage current is approximately sinusoidal, while after the accelerated aging experiment, there is a significant harmonic distortion in the leakage current. In addition, the leakage current amplitude increases gradually with the extension of the aging time, and the amplitude increases by 9.09%, 16.36%, and 38.18% after 12, 24, and 48 days of aging, respectively, compared with that of the unaged sample.

## 3. Time–Frequency Domain Analysis and Multidimensional Characteristic Parameter Extraction of Leakage Current

### 3.1. Time–Frequency Domain Analysis of Leakage Current

In order to further extract the information contained in the leakage current, the leakage current was analyzed using the time–frequency analysis method. Dragomiretskiy et al. [20] proposed a variational mode decomposition (VMD) as a variational mode-based signal processing method that could decompose the signal *x(t)* into a sequence of intrinsic mode function (*IMF(t)*) and residuals (res(t)). The original signal *x(t)* is the sum of *IMF(t)* and *res(t)*, as shown in Equation (1) [21].

(1) $$x(t)=\sum_{i=1}^{k}IMF_{i}(t)+res(t)$$

**Figure 6:**
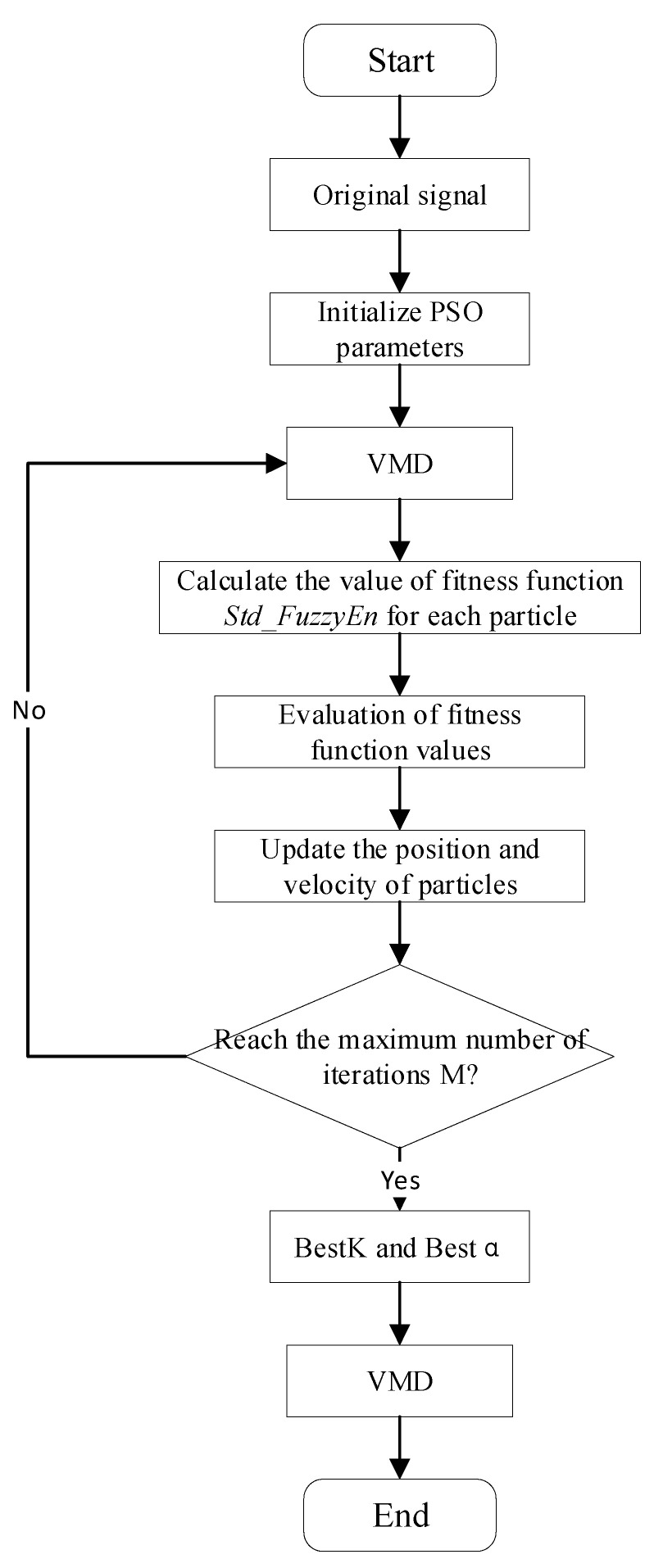
The flow chart of PSO–VMD algorithm.

Before performing the VMD on the signal, two parameters, the modal number *K* and the quadratic penalty factor *α*, need to be preset, where *K* determines the number of IMFs obtained by VMD, and *α* determines the bandwidth of each IMF. *K* and *α* have a significant impact on the VMD results, so this paper used particle swarm optimization (PSO) to find the optimal *K* and *α* in the VMD. The flow chart of the PSO–VMD algorithm is shown in Figure 6. The fitness function was the standard deviation of the fuzzy entropy of a set of IMFs obtained after the VMD, and its expression is shown in Equation (2). The specific procedure for calculating the fuzzy entropy is given by Reference [22]. The standard deviation can reflect the degree of data dispersion, and the fitness function is minimized when the optimal combination of *K* and *α* is obtained.

(2) $$\left\{ \begin{array}{l} Fitness(K,\alpha )=\sqrt{\frac{1}{K}\sum_{i=1}^{K}{\left(FuzzyEn_{i}-\mu \right)}^{2}}\\ \mu =\frac{1}{K}\sum_{i=1}^{K}FuzzyEn_{i} \end{array}\right.$$

**Figure 7:**
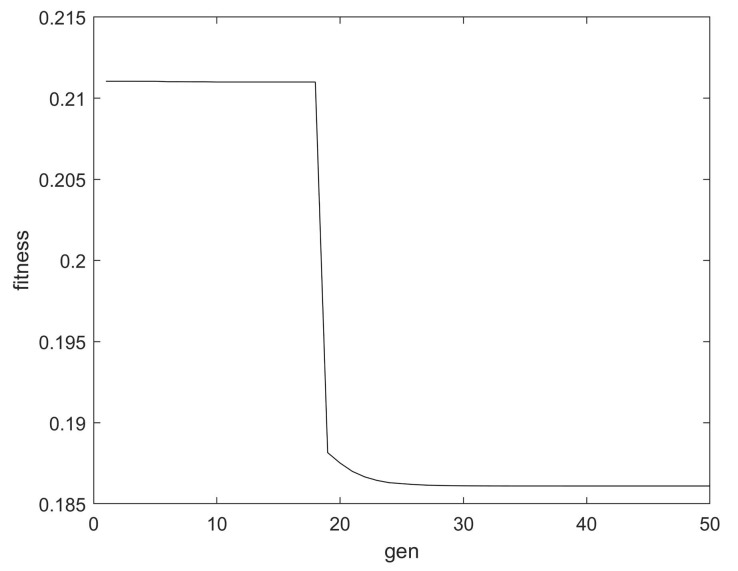
The fitness in the optimization process.

We set the range of *K* to 5~30 and the range of *α* to 1500~6000 and executed the optimization process. Figure 7 illustrates the change process of the fitness of the leakage current after aging for 12 days. From the figure, it can be seen that the fitness curve shows a trend of first decreasing and then converging, and the convergence is completed in the 35th generation. The optimal fitness was 0.186103, and the *K* and *α* at that time were 11 and 5070.33, respectively.

After the VMD, *K* IMF components can be obtained, and the instantaneous frequency and instantaneous amplitude of IMF components can be obtained by applying the Hilbert transform on the IMF components. For the time signal *x(t)*, its Hilbert transform expression [23] is

(3) $$\overset{\wedge }{x}(t)=H[x(t)]=\frac{1}{\pi }\int_{-\infty }^{+\infty }\frac{x(t)}{t-\tau }d\tau$$

**Figure 8:**
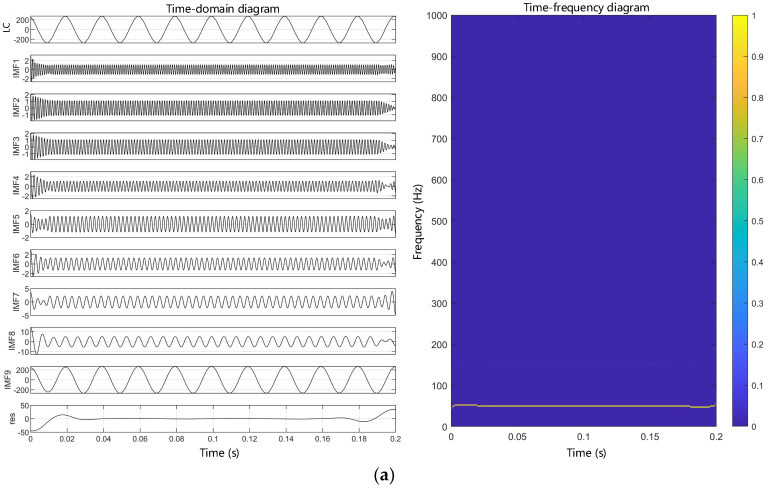

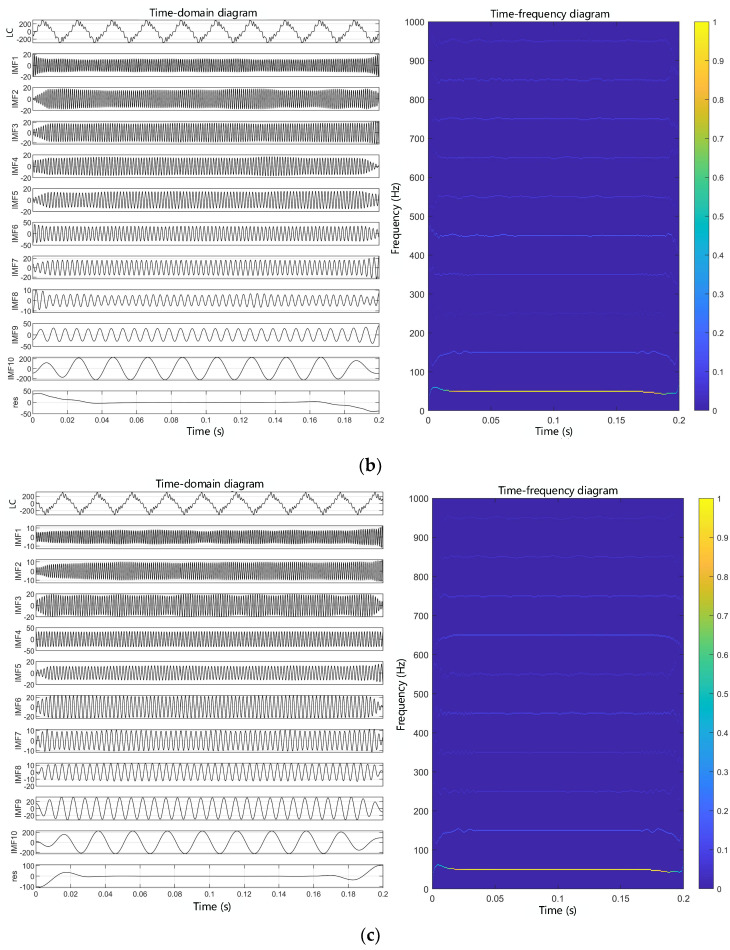

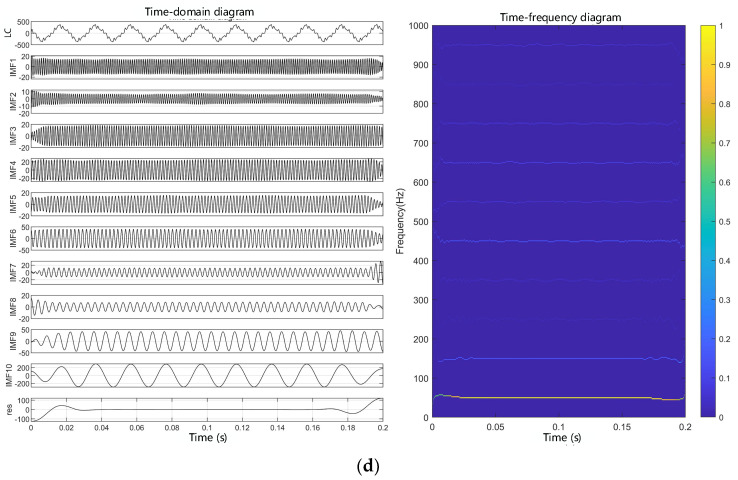
The time-frequency distribution of leakage current. (**a**) Unaged; (**b**) aging for 12 days; (**c**) aging for 24 days; (**d**) aging for 48 days.

The time-domain diagram and the time-frequency diagram of the leakage currents signal after the VMD are shown in Figure 8. The time-domain diagram shows that each IMF obtained after the VMD is a sinusoidal wave, and the time-frequency diagram further illustrates that these IMFs are actually fundamental and odd harmonics, so that the leakage currents is composed of a fundamental and a series of odd harmonics. As aging proceeds, the amplitude of each odd harmonic increases compared to the unaged sample, which can indicate that there is a certain connection between aging and harmonic generation.

### 3.2. Harmonic Selection and Signal Reconstruction

From the analysis in the previous section, the thermal aging cable leakage currents has a series of odd harmonics, which hold information about the cable’s thermal aging condition. A feature extraction can calculate and select some complete and nonredundant feature quantities from the leakage current, which can effectively indicate the thermal aging condition of cables.

Before feature extraction, the degree of correlation between each order of odd harmonics and the thermal aging state of the cable was evaluated to screen out specific harmonics that were more sensitive to changes in the thermal aging state. In this paper, Pearson’s correlation coefficient ***ρ*** and the harmonic current content rate *HRI* were used as evaluation indexes.

The expression of Pearson’s correlation coefficient ***ρ*** is [24]

(4) $$\rho_{X,Y}=\frac{\sum_{i=1}^{n}\left(X_{i}-\overset{-}{X}\right)\left(Y_{i}-\overset{-}{Y}\right)}{\sqrt{\sum_{i=1}^{n}{\left(X_{i}-\overset{-}{X}\right)}^{2}\sqrt{\sum_{i=1}^{n}{\left(Y_{i}-\overset{-}{Y}\right)}^{2}}}}$$

where ***ρ***_*X,Y*_ is the correlation coefficient between series *X* and *Y*, which reflects the degree of linear correlation between series *X* and *Y*.

The expression of the harmonic current content rate *HRI_h_* is

(5) $$HRI_{h}=\frac{I_{h}}{I_{1}}\times 100\%$$

where *I_h_* is the effective value of the h-order harmonic current, *I*_1_ is the effective value of the fundamental current, and *HRI* reflects the content of the harmonics of each order.

Table 1 shows the calculation results of ***ρ*** and *HRI* between each order of harmonics and leakage current. As can be seen from the table, for the unaged cable, the ***ρ*** of the 150 Hz harmonic is the highest, but it is only 0.0273, and its *HRI* is 2.0761%. With the increasing of harmonic order, the ***ρ*** and *HRI* gradually decrease. For the cable after aging for 12 days, the harmonics with a higher ***ρ*** and *HRI* are 450 Hz, 150 Hz, and 650 Hz in order; for cables with 24 and 48 days of thermal aging, they are 650 Hz, 150 Hz, and 450 Hz and 150 Hz, 450 Hz, and 650 Hz, respectively.

From the above analysis, it can be seen that the harmonics that are sensitive to the thermal aging state were at 150 Hz, 450 Hz, and 650 Hz. In this paper, the harmonics of these three frequencies were superimposed to obtain the reconstructed signal, and the features of the reconstructed signal were extracted.

### 3.3. Multidimensional Characteristic Parameter Extraction

In this section, for the leakage current data of the unaged cable and the cable after aging for 12 days, 24 days, and 48 days, 500 groups of reconstructed signals *x(i)*, *i* = 1, 2, …, *N* (*N* = 4000) were obtained, respectively, according to the above method. The time-domain, frequency-domain, and relative energy and the sample entropy characteristic parameters of these reconstructed signals were calculated, and the variation and distribution of these characteristic parameters were analyzed by using a statistical graph.

(1) Time-domain characteristic parameters

The time-domain characteristic parameters are shown in Table 2 [25].

**Figure 9:**
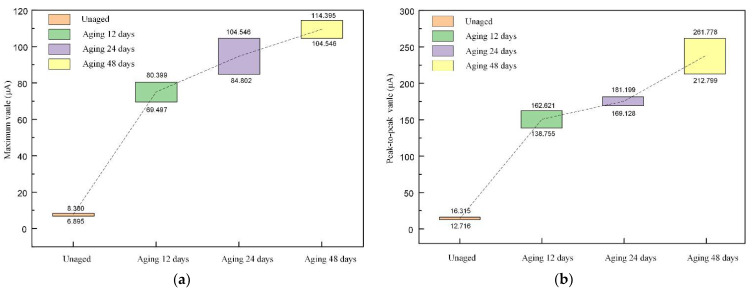
The distribution of dimensioned parameters in the time domain. (**a**) Maximum value; (**b**) peak-to-peak value.

For the reconstructed signal, the maximum value, peak-to-peak value, waveform factor, peak factor, pulse factor, and margin factor were extracted, where the maximum value and peak-to-peak value were dimensioned characteristic parameters, and their distributions are shown in Figure 9. From the figure, it can be seen that thermal aging makes the maximum and peak-to-peak values of the reconstructed signals increase significantly, and the degree of increase is also greater with the increase of the aging time. In addition, thermal aging makes their distribution range larger.

**Figure 10:**
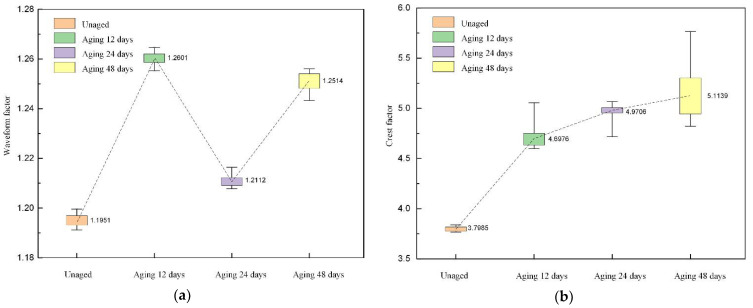

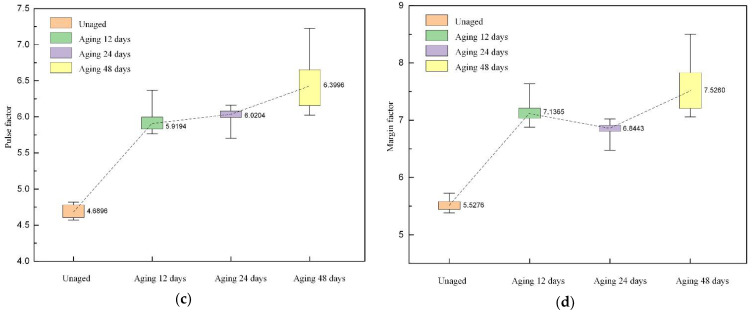
The distribution of dimensionless parameters in the time domain. (**a**) Waveform factor; (**b**) crest factor; (**c**) pulse factor; (**d**) margin factor.

The distribution of the waveform factor, peak factor, pulse factor, and margin factor of the reconstructed signal is shown in Figure 10. It can be seen from the figure that the waveform factor shows an increasing-decreasing-increasing trend as the aging time increases, while the peak factor, pulse factor, and margin factor show an increasing trend in fluctuation.

(2) Frequency-domain characteristic parameters

First, the power spectrum *P(m)* of the reconstructed signal was calculated, *m* = 1, 2, …, *M*, *M* was the number of spectral lines, and then the mean frequency, standard deviation frequency, center of gravity frequency, and root-mean-square frequency of the reconstructed signal were calculated, and their expressions are given in Table 3 [26]. The mean frequency reflects the magnitude of the power spectrum energy, the standard deviation frequency describes the degree of dispersion or concentration of the spectrum, while the center of gravity frequency and root-mean-square frequency reflect the variation of the main frequency band position.

**Figure 11:**
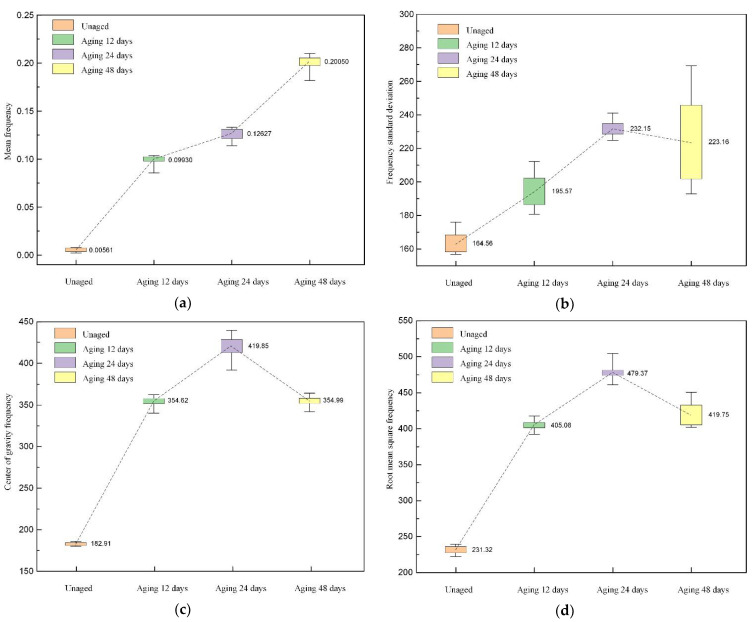
The distribution of frequency-domain parameters. (**a**) Mean frequency; (**b**) frequency standard deviation; (**c**) center of gravity frequency (**d**) root-mean-square frequency.

As can be seen in Figure 11, thermal aging causes the frequency-domain parameters of the reconstructed signal to increase. The increase of the mean frequency indicates that the power spectrum energy of the reconstructed signal increases, and the increase of the frequency standard deviation indicates that the frequency distribution of the reconstructed signal tends to be dispersed. The changes in the center of gravity frequency and root-mean-square frequency indicate that the thermal aging shifts the position of the main band of the reconstructed signal to the high-frequency direction.

(3) Relative energy and sample entropy

The expression of the relative energy *RE_k_* is

(6) $$RE_{k}=\frac{E_{k}}{E_{0}}\times 100\%$$

where *E_k_* is the energy of the reconstructed signal after 12, 24, and 48 days of aging, and *E*_0_ is the energy of the reconstructed signal that the unaged.

The sample entropy of the reconstructed signal was also calculated, which reflects the complexity of the reconstructed signal. The procedure for calculating the sample entropy can be obtained from the reference [27]. The value of the sample entropy is related to the values of the pattern dimension m and the similarity tolerance r. The results of previous studies have shown [28] that the sample entropy calculated by taking m = 1 or m = 2 and r = 0.1–0.25 Std (Std is the standard deviation of the original data) has more reasonable statistical properties. Therefore, we set m = 2 and r = 0.2 Std.

**Figure 12:**
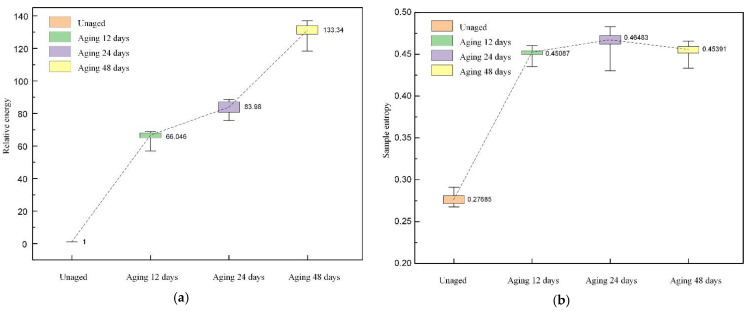
The distribution of relative energy and sample entropy. (**a**) Relative energy; (**b**) sample entropy.

The distributions of the relative energy and sample entropy are shown in Figure 12. From the figure, it can be seen that the relative energy of the reconstructed signal gradually increases with the extension of the thermal aging time, and the two show a positive correlation, which indicates that the thermal aging drives the generation and development of harmonics. In addition, the sample entropy of the reconstructed signal becomes larger after thermal aging, which indicates that thermal aging makes the reconstructed signal sequence disorder increase.

## 4. Conclusions

In this paper, cable samples were fabricated and accelerated aging experiments were conducted, test circuits were built, and the dielectric loss factor and leakage current tests were performed on cables under different aging times. Further, a time–frequency analysis of the leakage current signal was carried out, focusing on the variation of harmonics in the leakage current, and the multidimensional characteristic parameters of sensitive harmonics were extracted and analyzed. The main conclusions obtained were as follows:

(1) The capacitance of the cable sample, the dielectric loss factor, and the amplitude of the leakage current of the cable sample gradually increased with the extension of the aging time. This indicated that the thermal aging made the dielectric properties of XLPE insulation deteriorate and the losses increase.
(2) Thermal aging drove the generation of odd harmonic components in the cable leakage current and the amplitude of these harmonic components correlated with the degree of thermal aging. Among the odd harmonic components, the harmonics at 150 Hz, 450 Hz, and 650 Hz were more sensitive to the changes in the degree of thermal aging.
(3) Thermal aging increased the relative energy and power spectrum energy of the sensitive harmonics and increased the disorder of the sensitive harmonic sequence.

## Figures and Tables

**Table 1:** The ***ρ*** and *HRI* between each order of harmonics and leakage current.

Harmonic Frequency (Hz)	Unaged		Aging 12 Days		Aging 24 Days		Aging 48 Days	
	*ρ*	*HRI* (%)	*ρ*	*HRI* (%)	*ρ*	*HRI* (%)	*ρ*	*HRI* (%)
150	0.0273	2.0761	0.1348	13.2395	0.1445	14.4843	0.1478	14.7071
250	0.0127	0.8584	0.0363	2.6265	0.0586	5.8067	0.0379	3.0394
350	0.0081	0.4936	0.0705	7.5498	0.0469	4.6530	0.0496	4.6078
450	0.0064	0.4367	0.1363	14.8848	0.0984	10.7732	0.1334	14.4038
550	0.0069	0.4311	0.0621	6.6498	0.0540	5.7957	0.0513	5.3084
650	0.0051	0.4188	0.0770	8.4036	0.1424	15.8693	0.0852	9.0979
750	0.0049	0.4198	0.0648	7.0084	0.0784	8.5950	0.0600	6.4019
850	0.0057	0.4246	0.0686	7.4309	0.04132	4.4566	0.0260	2.5617
950	-	-	0.0508	5.5170	0.0329	3.5663	0.0497	5.2813

**Table 2:** The time-domain characteristic parameters.

Characteristic Parameter	Expression	Characteristic Parameter	Expression
Mean value	$\bar{X}=\frac{1}{N}\sum_{i=1}^{N}x(i)$	Root-mean-square value	$X_{\mathrm{rms}}=\sqrt{\frac{1}{N}\sum_{i=1}^{N}x(i)}$
Variance	$\sigma^{2}=\frac{1}{N-1}\sum_{i=1}^{N}{\left(x(i)-\bar{X}\right)}^{2}$	Square root amplitude	$X_{r}={\left[\frac{1}{N}\sum_{i=1}^{N}\sqrt{\left|x(i)\right|}\right]}^{2}$
Absolute mean value	$\left|\bar{X}\right|=\frac{1}{N}\sum_{i=1}^{N}\left|x(i)\right|$	Crest factor	$C_{f}=\frac{X_{\max}}{X_{\mathrm{rms}}}$
Maximum value	$X_{\max}=\max\left\{x(i)\right\}$	Waveform factor	$S_{f}=\frac{X_{rms}}{\left|\bar{X}\right|}$
Minimum value	$X_{\min}=\min\left\{x(i)\right\}$	Pulse factor	$I_{f}=\frac{X_{\max}}{\left|\bar{X}\right|}$
Peak-to-peak value	$X_{\mathrm{pp}}=X_{\max}-X_{\min}$	Margin factor	$CL_{f}=\frac{X_{\max}}{X_{r}}$

**Table 3:** The frequency-domain characteristic parameters.

Characteristic Parameter	Expression
Mean frequency	$\overset{-}{f}=\frac{1}{M}\sum_{m=1}^{M}P(m)$
Standard deviation frequency	$f_{\sigma }=\sqrt{\sum_{m=1}^{M}\left\{{\left[f_{k}-f_{c}\right]}^{2}\times P(m)\right\}/m}$
Center of gravity frequency	$f_{c}=\frac{\sum_{m=1}^{M}\left[f_{k}\times P(m)\right]}{\sum_{m=1}^{M}P(m)}$
Root-mean-square frequency	$f_{RMS}=\sqrt{\sum_{m=1}^{M}\left[{f_{k}}^{2}\times P(m)\right]/\sum_{m=1}^{M}P(m)}$

## Data Availability

The data presented in this study are available on request from the corresponding author. The data are not publicly available due to privacy reasons.
